# *Bifidobacterium breve* MRx0004 protects against airway inflammation in a severe asthma model by suppressing both neutrophil and eosinophil lung infiltration

**DOI:** 10.1038/s41598-018-30448-z

**Published:** 2018-08-13

**Authors:** Emma J. Raftis, Margaret I. Delday, Philip Cowie, Seánín M. McCluskey, Mark D. Singh, Anna Ettorre, Imke E. Mulder

**Affiliations:** 1 4D Pharma Research Ltd, Life Science Innovation Building, Cornhill Road, Aberdeen, AB25 2ZS United Kingdom; 2 Institute of Medical Sciences, Foresterhill, University of Aberdeen, Aberdeen, AB25 2ZD United Kingdom

## Abstract

Asthma is a phenotypically heterogeneous disease. In severe asthma, airway inflammation can be predominantly eosinophilic, neutrophilic, or mixed. Only a limited number of drug candidates are in development to address this unmet clinical need. Live biotherapeutics derived from the gut microbiota are a promising new therapeutic area. MRx0004 is a commensal *Bifidobacterium breve* strain isolated from the microbiota of a healthy human. The strain was tested prophylactically and therapeutically by oral gavage in a house dust mite mouse model of severe asthma. A strong reduction of neutrophil and eosinophil infiltration was observed in lung bronchoalveolar lavage fluid following MRx0004 treatment. Peribronchiolar and perivascular immunopathology was also reduced. MRx0004 increased lung CD4^+^CD44^+^ cells and CD4^+^FoxP3^+^ cells and decreased activated CD11b^+^ dendritic cells. Cytokine analysis of lung tissue revealed reductions of pro-inflammatory cytokines and chemokines involved in neutrophil migration. In comparison, anti-IL-17 antibody treatment effectively reduced neutrophilic infiltration and increased CD4^+^FoxP3^+^ cells, but it induced lung eosinophilia and did not decrease histopathology scores. We have demonstrated that MRx0004, a microbiota-derived bacterial strain, can reduce both neutrophilic and eosinophilic infiltration in a mouse model of severe asthma. This novel therapeutic is a promising next-generation drug for management of severe asthma.

## Introduction

Asthma is an umbrella term for a highly heterogeneous disease with clinical presentations ranging from mild to severe. It is a chronic inflammatory lung disease characterised by recurrent, reversible airway obstruction and increased bronchial hyper-responsiveness. Asthma affects an estimated 330 million people worldwide^1^, and its incidence will rise by an additional 100 million people by 2025^2^. The socio-economic costs of asthma are substantial due to the significant healthcare burden associated with the condition^3,4^. Between 5–10% of asthma patients have a severe form of the disease which is refractory to steroid treatment and whose symptoms cannot be controlled despite the application of high-intensity treatments^5^. Severe asthma accounts for more than 60% of asthma-associated healthcare costs^6^.

Endotypes of severe asthma are associated with distinct pathophysiological mechanisms characterised by molecular phenotypes, associated biomarkers and differential responses to therapy^7,8^. The endotypes are increasingly stratified by the extent of immune cell infiltration, which is often either predominantly eosinophilic or neutrophilic, but can present as a mixed eosinophilic/neutrophilic inflammatory pattern^9–13^. Broad divisions have been drawn based on observed levels of T_H_2 eosinophilic inflammation, which include T_H_2-high and T_H_2-low/non-T_H_2 phenotypes^14,15^. Endotype-based patient stratification has proven crucial to the effective clinical application of antibody-derived therapies targeting T_H_2 cytokines in severe eosinophilic asthma^16–19^. Similar approaches targeting T_H_1 or T_H_17 pathway effectors such as CXCR2 and IL-17 have shown some promise in the treatment of T_H_2-low, neutrophilic asthma^20–22^. However, results remain inconclusive and there are currently no approved therapeutics for this group of severe asthma patients^23^.

Due to the multifactorial and heterogeneous nature of severe asthma, blocking a single mediator is unlikely to be effective across multiple phenotypes. Furthermore, different inflammatory pathways are often reciprocally regulated, whereby neutralization of one cytokine can lead to activation of the opposing pathway^24^. The limitations of current treatment approaches highlight the need for new therapeutics that target underlying immune responses associated with severe asthma to provide greater disease control and increased efficacy.

There is a wealth of evidence which illustrates the pivotal role of the gut microbiota in shaping aspects of both innate and adaptive immune responses^25,26^. The mechanisms by which gut bacteria impact immunological pathways are steadily being elucidated and include specific expansion of regulatory T^27–29^ and B cells^30,31^, induction of T_H_17 responses^32,33^ and inhibition of invariant natural killer T cells^34,35^. While the functional relationship between the intestinal microbiota and chronic respiratory diseases is still poorly understood, altered abundance of *Bifidobacterium* species has been associated with long-term asthma^36^, atopic diseases^37,38^ and cystic fibrosis^39^. Allergic T_H_2-low asthma studies have shown that oral delivery of *Bifidobacterium* strains has the potential to alleviate disease symptoms^40–42^. Therefore, the gut microbiota provides a promising reservoir of novel therapeutic potential for asthma.

Here, we provide evidence of the efficacy of MRx0004, a *Bifidobacterium breve* strain isolated from the gut microbiome of a healthy human, in a steroid-resistant mouse model of severe asthma. Daily oral dosing with MRx0004, using either a prophylactic or therapeutic intervention strategy, reduced lung inflammation by decreasing infiltration of both neutrophils and eosinophils. MRx0004 elicited a general anti-inflammatory effect on lung immune populations including regulatory T cells and dendritic cells (DCs). Interestingly, anti-IL-17 antibody treatment reduced neutrophilic infiltration, but exacerbated lung eosinophilia and inflammatory histopathology scores. MRx0004 is a promising next-generation therapeutic with the potential to affect the different inflammatory cell types involved in severe, steroid-resistant asthma.

## Results

### The microbiota-derived bacterial strain MRx0004 reduces total BALF cell count, neutrophil and eosinophil lung infiltration in a severe asthma model

We investigated the effects of MRx0004, a *Bifidobacterium breve* strain, on airway inflammation in a murine severe steroid-resistant asthma model. C57BL/6 mice were sensitized with house dust mite (HDM) in Complete Freund’s Adjuvant (CFA) by subcutaneous injection at day (D) D0 and D7, and then challenged intranasally with HDM on D14, D15, D16 and D17 (See Supplementary Fig. S1). Animals were prophylactically dosed by oral gavage with MRx0004 for two weeks and throughout the disease phase. An anti-IL-17 antibody comparator group was included (intraperitoneal injection at D13, D15 and D17), as the role of IL-17 family members in promoting neutrophilic inflammation is well established, with correlations between lung IL-17 levels and disease severity in neutrophilic asthma (see review^43^). On D18, animals were sacrificed followed by collection of bronchoalveolar lavage fluid (BALF) and lung tissue for subsequent analysis. No morbidity or mortality was noted in any of the treatment groups during the study.

**Figure 1:**
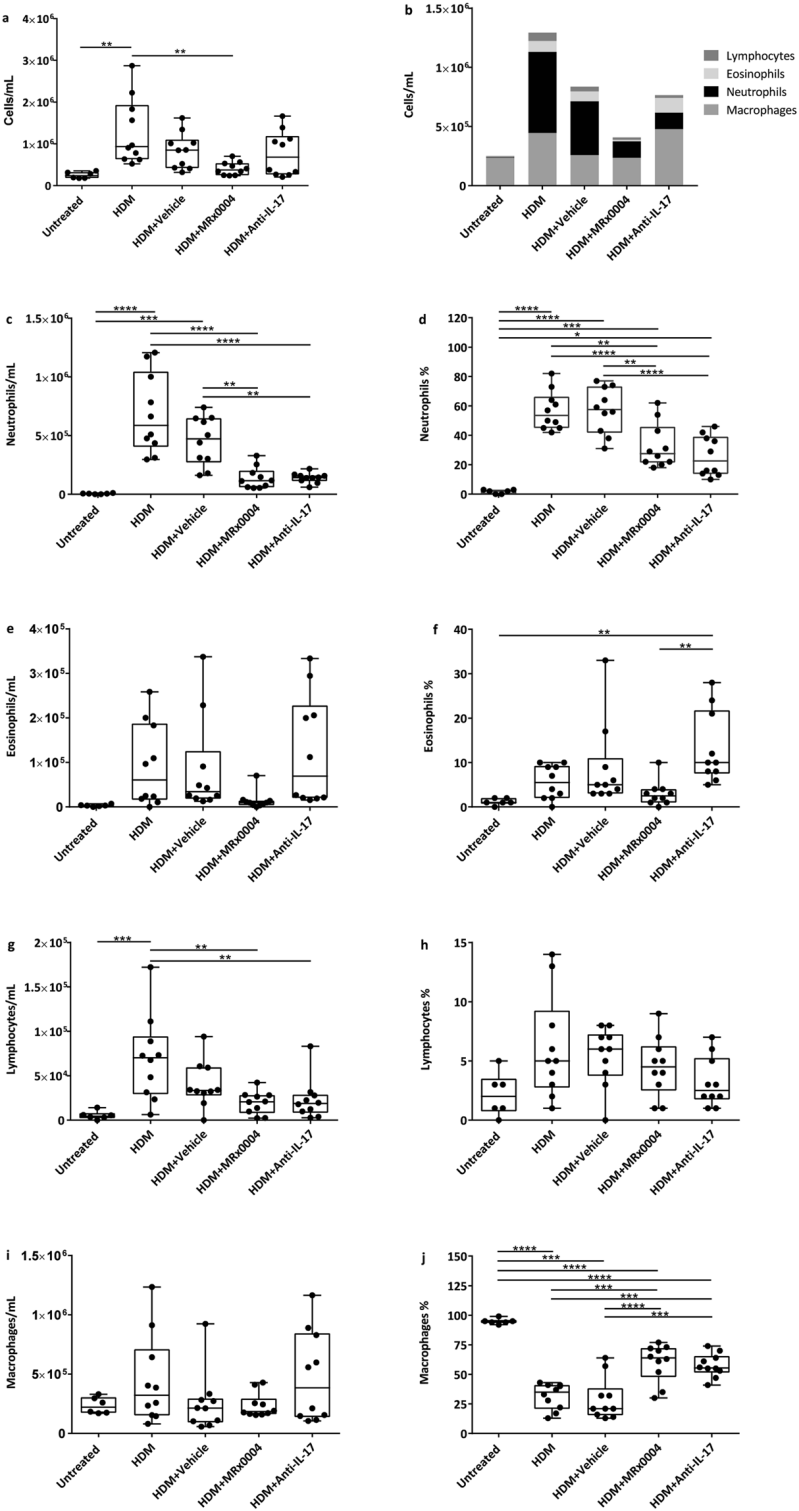
Effect of MRx0004 on BALF cell counts. Bronchoalveolar lavage fluid (BALF) cell counts of mice exposed to HDM, and treated with MRx0004, anti-IL-17 or vehicle, with samples collected 24 h after final exposure. (**a**) Total cell count; (**b**) total cell counts presented as different cell subtypes; (**c**) total neutrophils; (**d**) % neutrophils; (**e**) total eosinophils; (**f**) % eosinophils; (**g**) total lymphocytes; (**h**) % lymphocytes; (**i**) total macrophages; (**j**) % macrophages. Results are shown as box and whisker plots with individual data points (n = 6 for untreated, n = 10 for all other groups). Capped bars illustrate the maximum and minimum data points within each treatment group. *p < 0.05, **p < 0.01, ***p < 0.001, ****p < 0.0001 between treatment groups.

BALF was examined to quantify the extent of inflammatory cell infiltration (Fig. 1). The key parameter for assessment of efficacy was the number of neutrophils recruited into the airways. HDM-sensitized mice had a significantly higher number of BALF cells/mL than untreated mice (Fig. 1a,b), accompanied by severe inflammation characterized by a large neutrophilic influx (Fig. 1c and d). MRx0004 significantly reduced the total number of BALF cells compared to HDM alone (Fig. 1a,b) and was highly efficacious in alleviating the magnitude of the neutrophilic inflammatory response. Importantly, both total number and percentage of neutrophils were significantly reduced by MRx0004 compared to HDM and HDM+ vehicle (referred to as vehicle group below) (Fig. 1c and d). Anti-IL-17 treatment also significantly reduced neutrophilic infiltration. While eosinophil infiltration in this model is known to be low compared to neutrophil infiltration, MRx0004 also reduced (albeit not significantly) the number and percentage of BALF eosinophils (Fig. 1e and f). This was significantly lower than the anti-IL-17 group, which showed increased proportions of eosinophils compared to the other treatment groups (Fig. 1f). A significant increase in lymphocyte numbers was observed in the HDM group compared to untreated animals, but not in either HDM+ MRx0004 (referred to as MRx0004 group below) or HDM+ anti-IL-17 (referred to as anti-IL-17 group below) (Fig. 1g). The reduced neutrophil and eosinophil lung infiltration after MRx0004 treatment was accompanied by an increased proportion of BALF macrophages (Fig. 1i and j). Anti-IL-17 treatment also significantly increased BALF macrophage percentage compared to HDM and vehicle animals (Fig. 1j).

### Reduction of neutrophil and eosinophil numbers by MRx0004 is associated with lower lung inflammatory scores

**Figure 2:**
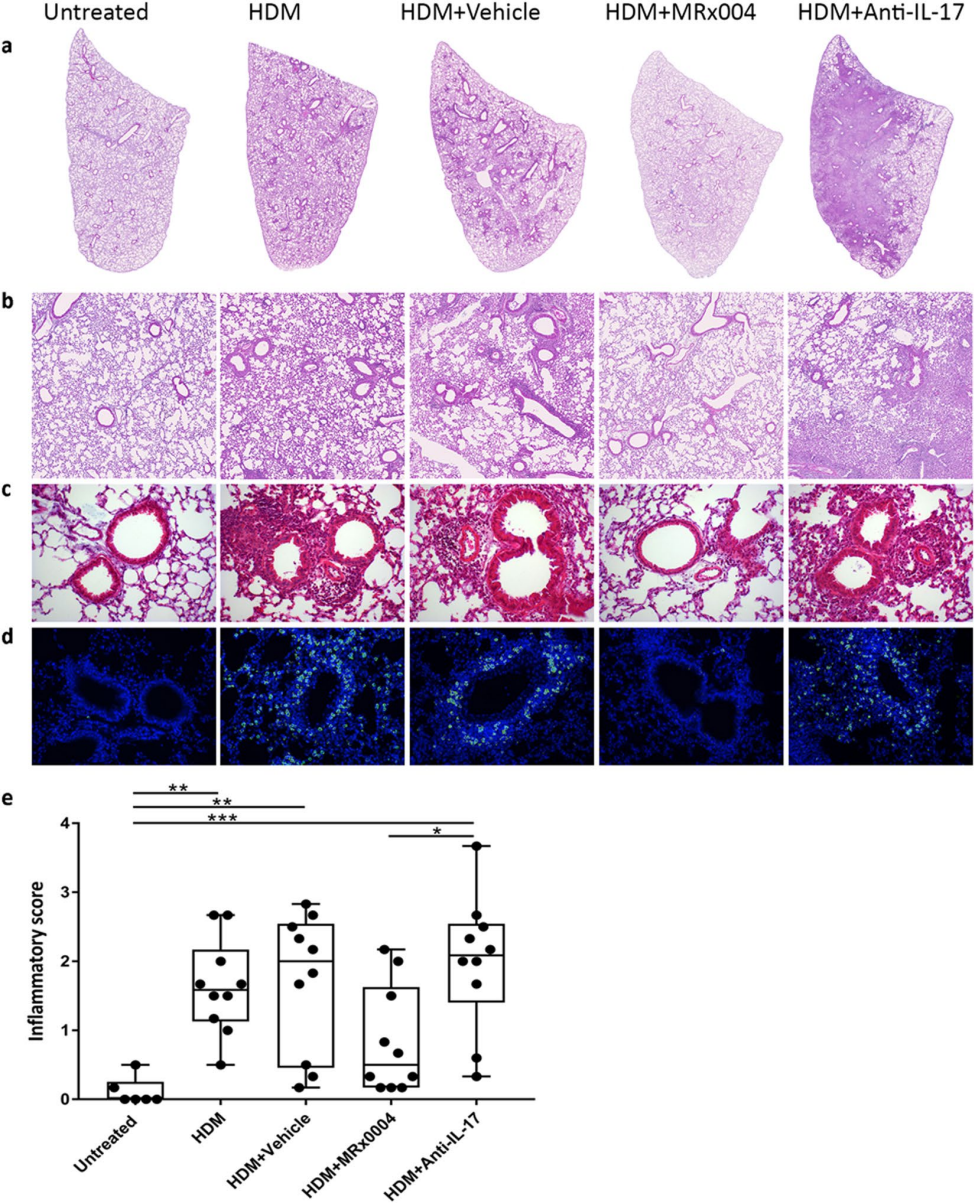
Effect of MRx0004 on lung histopathology and inflammatory scores. Histopathological analysis of lungs of mice exposed to HDM, and treated with MRx0004, anti-IL-17 or vehicle, with samples collected 24 h after final exposure. (**a**) Representative scans (original magnification x25) of H&E stained FFPE sections of left lungs; (**b**) digital zoom of the lung mid region; (**c**) representative images (original magnification x200) for histopathology scoring; (**d**) immuno-labelled CD4^+^ cells (green), double-labelled with DAPI (blue); (**e**) mean inflammatory scores. Results are shown as box and whisker plots with individual data points (n = 6 for untreated, n = 10 for all other groups). Capped bars illustrate the maximum and minimum data points within each treatment group. *p < 0.05, **p < 0.01, ***p < 0.001 between treatment groups.

Histological analysis for both peribronchiolar and perivascular inflammatory infiltration was performed on Haematoxylin & Eosin-stained lung sections. The extent of infiltrating cells was evident in both representative scans of whole lung sections and in higher-magnification images (Fig. 2a–c) and was observed around both the bronchioli and the blood vessels (Fig. 2c). MRx0004 treatment strongly reduced peribronchiolar and perivascular inflammatory cell infiltration, resulting in lung histological appearance similar to that of untreated animals. This was in contrast to the inflammatory infiltrate in anti-IL-17-treated animals, which was most comparable to the HDM and vehicle groups. Immunohistochemistry staining showed high-level infiltration of CD4^+^ cells in the HDM and vehicle treated groups, with anti-IL-17 treated mice showing an even greater degree of CD4^+^ positive cells (Fig. 2d). The untreated and MRx0004 groups had very low numbers of CD4^+^ stained cells compared to the other groups.

Both the HDM and vehicle groups showed significantly higher infiltration scores compared to untreated animals (Fig. 2e). Lung histopathological scores of MRx0004-treated animals were not significantly different from untreated animals. These data support the differential cell counts from the BALF that showed reduced cellular infiltrate (Fig. 2e). While anti-IL-17 treatment had strong effects on neutrophil BALF infiltration, it was unsuccessful in reducing lung inflammatory scores.

### MRx0004 treatment reduces pro-inflammatory cytokine production in the lung

**Figure 3:**
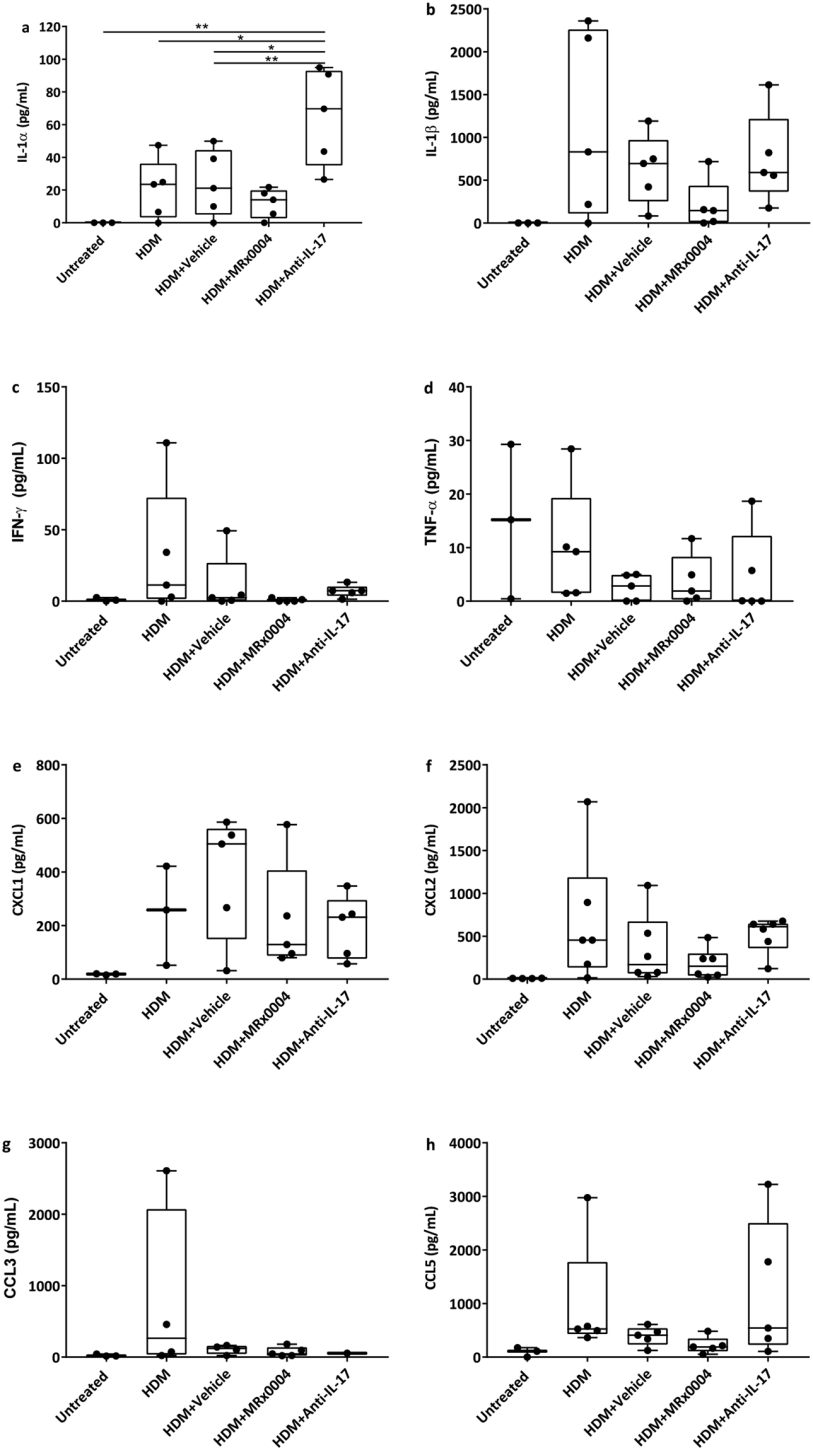
Impact of MRx0004 on lung inflammatory mediators. Cytokine and chemokine levels in right lungs of mice exposed to HDM, and treated with MRx0004, anti-IL-17 or vehicle, with samples collected 24 h after final exposure. (**a**) IL-1α; (**b**) IL-1β; (**c**) IFN-γ; (**d**) TNF-α; (**e**) CXCL1; (**f**) CXCL2; (**g**) CCL3; (**h**) CCL5. Results are shown as box and whisker plots with individual data points (n = 3 for untreated, n = 5 for all other groups). Capped bars illustrate the maximum and minimum data points within each treatment group. *p < 0.05, **p < 0.01, ***p < 0.001, ****p < 0.0001 between treatment groups.

Analysis of cytokine levels in the lung tissue showed that disease progression was associated with a trend towards increased IL-1α, IL-1β, IFN-γ, CXCL1 and CXCL2 in HDM and vehicle-treated animals (Fig. 3). While none of the data reached statistical significance, MRx0004 reduced the pro-inflammatory cytokines IL-1α and IL-1β and the chemokine CXCL2. Anti-IL-17 treated mice showed a significant increase in IL-1α compared to all other treatments.

### Therapeutic dosing of MRx0004 also reduces the neutrophil inflammatory response

**Figure 4:**
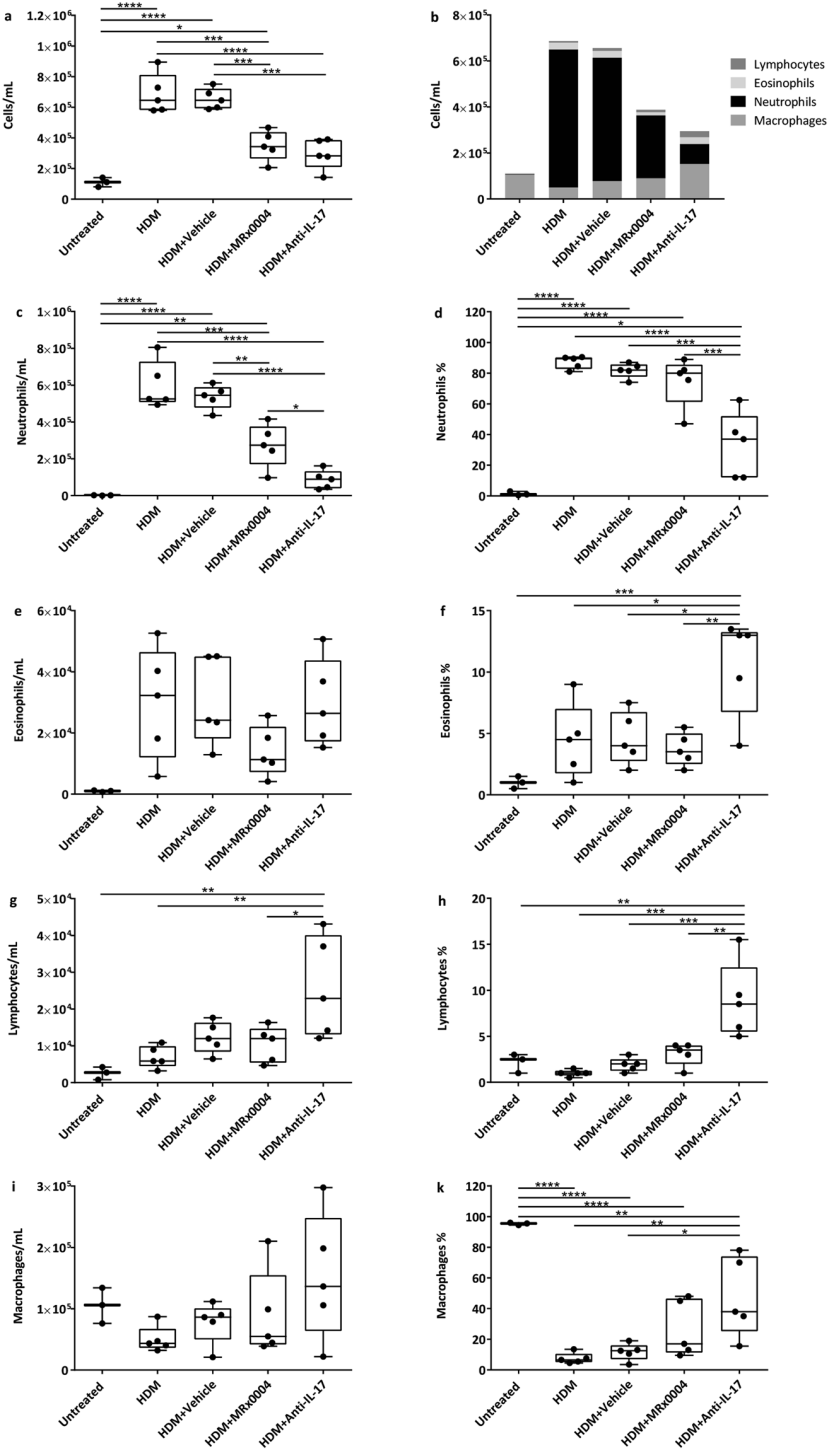
Effect of therapeutically-dosed MRx0004 on BALF cell counts. Bronchoalveolar lavage fluid (BALF) cell counts of mice exposed to HDM, and treated with MRx0004 (therapeutically), anti-IL-17 or vehicle, with samples collected 24 h after final exposure. (**a**) Total cell count; (**b**) total cell counts presented as different cell subtypes; (**c**) total neutrophils; (**d**) % neutrophils; (**e**) total eosinophils; (**f**) % eosinophils; (**g**) total lymphocytes; (**h**) % lymphocytes; (**i**) total macrophages; (**j**) % macrophages. Results are shown as box and whisker plots with individual data points (n = 3 for untreated, n = 5 for all other groups). Capped bars illustrate the maximum and minimum data points within each treatment group. *p < 0.05, **p < 0.01, ***p < 0.001, ****p < 0.0001 between treatment groups.

MRx0004 was tested using a therapeutic dosing strategy to determine whether it could prevent or limit airway inflammation during the active phase of the disease. Following initial sensitization with HDM on D0, animals were dosed daily with MRx0004 from D7 until D17. MRx0004 significantly reduced airway neutrophil infiltration compared to the HDM and vehicle groups (Fig. 4c). In contrast to prophylactic dosing however, this reduction could largely be explained by the reduced total number of BALF cells, as opposed to a shift in the proportions of different cell types (Fig. 4a,b). Again, a trend towards a reduction in eosinophil numbers in MRx0004-treated mice was observed with therapeutic dosing (Fig. 4e). As expected, anti-IL-17 treatment showed a large reduction in total number and percentage of neutrophils. This treatment significantly increased eosinophils, macrophages and lymphocytes in the BALF (Fig. 4e,g and i).

### MRx0004 reduces both the number and activation state of pulmonary T cells and dendritic cells

**Figure 5:**
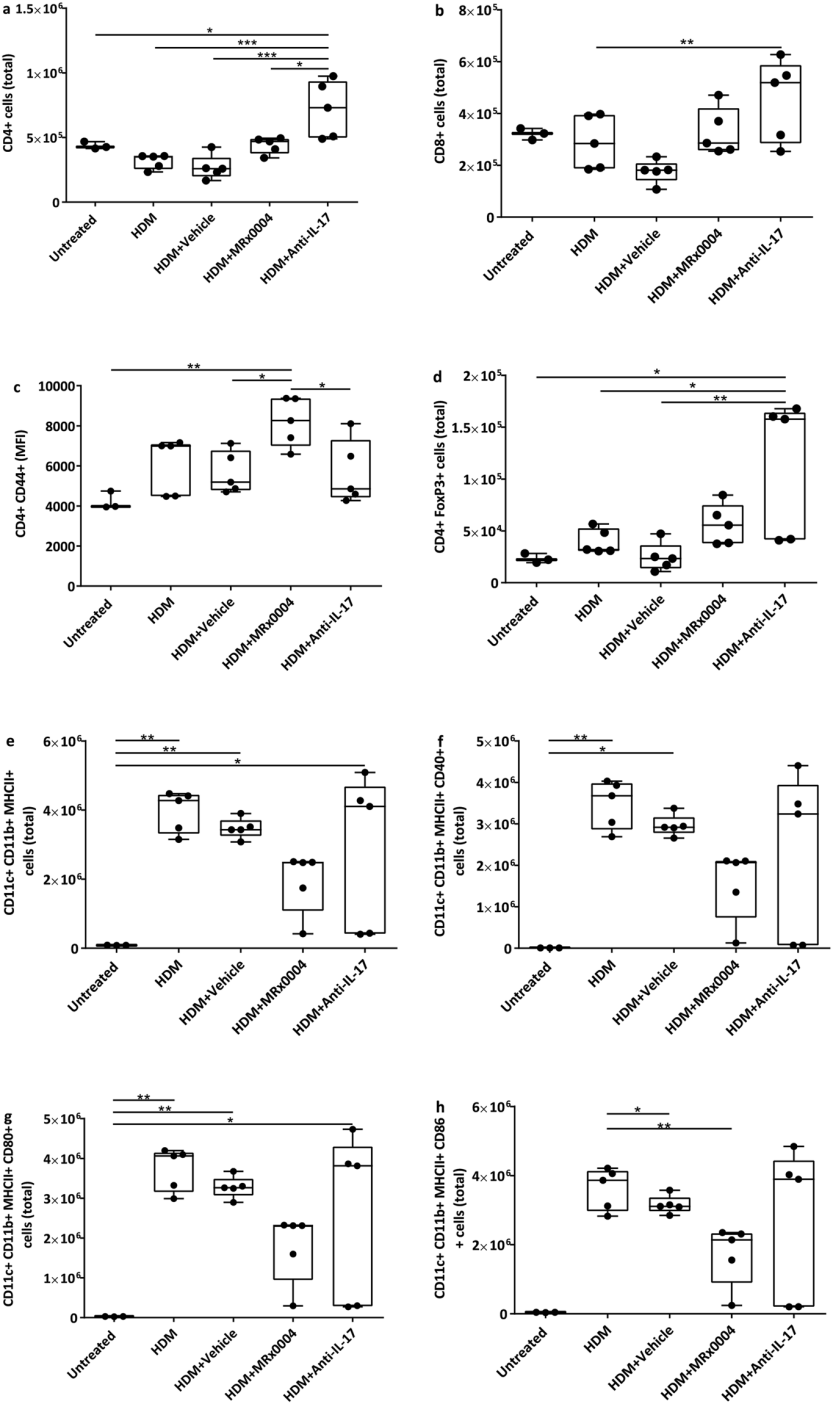
Impact of MRx0004 on T cell and dendritic cell numbers and activation status. Flow cytometry analysis of lung inflammatory infiltrates of mice exposed to HDM, and treated with MRx0004 (therapeutically), anti-IL-17 or vehicle, with samples collected 24 h after final exposure. (**a**) Total number of CD4^+^ cells; (**b**) total number of CD8^+^ cells; (**c**) the Mean Fluorescence Intensity (MFI) of the CD44 marker of activation on CD4^+^ and CD8^+^; (**d**) total number of CD4^+^FoxP3^+^ cells; (**e**) total number of CD11b^+^ cDCs; (**f**) total number of CD40^+^ cDCs; (**g**) total number of CD80^+^ cDCs; (**h**) total number of CD86^+^ cDCs. Results are shown as box and whisker plots with individual data points (n = 3 for untreated, n = 5 for all other groups). Capped bars illustrate the maximum and minimum data points within each treatment group. *p < 0.05, **p < 0.01, ***p < 0.001, ****p < 0.0001 between treatment groups.

Flow cytometry analysis of lung infiltrates was performed in the therapeutic dosing experiment to identify immune cell subsets involved in the protective mechanism of MRx0004. CD4^+^ T cells were significantly more abundant in the lungs of anti-IL-17 treated mice compared to all other treatment groups (Fig. 5a), while for MRx0004, the level was comparable to untreated animals. Anti-IL-17-treated mice also showed an increase in CD8^+^ T cells compared to the vehicle group (Fig. 5b). Only MRx0004 treatment increased the expression of the effector/memory T cell marker CD44 on these cells (Fig. 5c). A significant increase in CD4^+^ FoxP3^+^ regulatory T cell populations was observed in anti-IL-17 treated mice compared to HDM and vehicle groups (Fig. 5d). Treatment with MRx0004 also increased the total number of regulatory T cells compared to control groups, although this was not statistically significant. Expression of MHC II, CD40, CD80 and CD86 was used to identify activated dendritic cells. A trend towards reduced CD11b^+^ MHCII^+^ dendritic cell numbers was observed in the lungs of MRx0004-treated mice compared to HDM animals (Fig. 5e). A similar trend was apparent for the activation markers CD40 and CD80, with CD86 showing a significant reduction compared to HDM animals (Fig. 5f–h).

## Discussion

In this study we report that the candidate live biotherapeutic agent, *Bifidobacterium breve* MRx0004, significantly reduces disease pathology in a model of steroid-resistant severe asthma. HDM is a well-characterized allergen that can trigger allergic diseases such as asthma. C57BL/6 mice were sensitized to HDM in CFA to generate a steroid-insensitive neutrophilic and eosinophilic response, with similar inflammatory mediators to severe asthma in humans. Oral delivery of MRx0004 reduced airway inflammation in both prophylactically and therapeutically-treated animals. The strain was capable of preventing disease development as well as halting disease progression.

MRx0004 affected airway inflammation by strongly inhibiting neutrophilic and eosinophilic lung infiltration, and this resulted in a reduced total number of cells within the BALF compared to HDM-treated mice. Neutrophil infiltration in the lungs of severe asthma patients plays a functional role in disease pathogenesis^44^, and activated CD4^+^ T_H_17 cells promote neutrophilia through IL-17 production^45^. In this study, anti-IL-17 exclusively reduced the neutrophilic cell component in the BALF; however, this led to a concomitant increase in eosinophil numbers, in contrast to MRx0004 treatment.

Differences in disease severity were observed between the two animal experiments. While the first experiment showed a mixed neutrophilic/eosinophilic phenotype, the second experiment was more heavily skewed towards a stronger neutrophilic infiltration, with a lower proportion of eosinophils and macrophages. Interestingly, MRx0004 appeared more effective when the disease manifested both a neutrophilic and eosinophilic component, whereas anti-IL-17 showed highest efficacy on neutrophilic infiltrate. The reduced neutrophil and eosinophil lung infiltration observed in response to MRx0004 treatment was accompanied by an increased proportion of macrophages in the BALF. Alveolar macrophages form 90–95% of the cellular content in steady state^46^ and their increased proportions are thus an additional indication of the efficacy of MRx0004.

MRx0004 also had a striking effect on lung histopathology, as illustrated by the strong reduction of peribronchiolar and perivascular inflammatory infiltrate. The majority of MRx0004-treated animals showed low inflammatory scores similar to those of untreated animals. While anti-IL-17 antibody significantly reduced the numbers of neutrophils in the BALF, histopathological analysis of the lungs did not demonstrate an effect on infiltrating cells in the lung tissue. Other studies have similarly shown that, even though anti-IL-17 antibody treatment reduces BALF cellularity in HDM-induced asthma models, cell infiltration scores^47^ are not affected to the same degree. Increased airway inflammation was associated with an increase in CD4^+^ cells. This was particularly striking in the anti-IL-17 treated group. MRx0004-induced reduction of histopathology scores was reflected in a reduction in the abundance of CD4^+^ cells.

T_H_2 and T_H_17 inflammatory pathways are reciprocally regulated in asthma^24^. Neutralization of T_H_2 cytokines can result in increased T_H_17 cell populations and neutrophilic lung inflammation, while neutralization of T_H_17 responses can increase eosinophilia. Therapeutic targeting of T_H_2 or T_H_17 cytokines can thus amplify activity of the opposing pathway. However, neutralization of both types of cytokines protects mice from eosinophilia, mucus hyperplasia, and airway hyper-reactivity and abolishes neutrophilic inflammation^24^. While there have been some promising developments in the field, targeting specific pathways associated with either T_H_2-high or T_H_2-low disease can lead to undesirable side effects. Inhaled corticosteroid treatment to target eosinophilic asthma can also lead to airway neutrophilia^48,49^.

It has therefore been suggested that combination therapies targeting both pathways may maximize therapeutic efficacy across patient populations comprising both T_H_2 and T_H_17 endotypes^24^. As demonstrated in the current study, MRx0004 as a monotherapy can reduce the infiltration of both T_H_2 and T_H_17-associated granulocytes, indicating that it potentially reduces activation of both T helper pathways.

Efficacy of MRx0004 was reflected in a reduction in the levels of the lung inflammatory cytokines IL-1α, IL-1β and CXCL2, in particular compared to anti-IL-17. These pro-inflammatory mediators are known to be elevated in T_H_2-low asthmatic patients and although readouts failed to reach statistical significance in the current model, the suppression of these cytokines may provide insight into the mechanism by which MRx0004 elicits its effect. IL-1α (and IL-1β to a lesser extent) induces the production of CXCL1 and CXCL2, chemokines that have established roles in neutrophilic chemotaxis via CXCR1 and CXCR2^50–52^. A reduction in the levels of these cytokines and chemokines in MRx0004-treated animals may result in reduced neutrophil migration to the lungs. Previous studies have shown partial efficacy of an antagonist targeting CXCR2 in T_H_2-low, neutrophilic asthma patients^21,22^. Additionally, anti-IL-1β blocking antibodies have been shown to reverse lung pathology in steroid-resistant models of severe asthma^53^. Collectively, these data show the importance of IL-1/CXCL chemokine-mediated neutrophil migration in asthma.

There is increasing evidence linking the composition and function of the microbiome with susceptibility to and treatment of asthma^40,54,55^. To date, research has mostly focused on the relationship between gut bacteria and allergic T_H_2-driven asthma, with less attention paid to the severe, neutrophilic/eosinophilic asthma patient population. *Bifidobacterium* species induce distinct cytokine production patterns^56^ and can suppress allergic immune responses in a strain-specific manner^57–59^. Multiple studies by Sagar *et al*. demonstrated that *B*. *breve* M-16 V has strong anti-inflammatory properties and was capable of suppressing pulmonary airway inflammation and airway remodelling in a model of chronic allergic asthma^41,42^. Additionally, *B*. *breve* strains are capable of directing DCs and naïve CD4^+^ T cell polarization towards different effector or regulatory T cell subsets^41,42,56^. We therefore investigated the cellular dynamics of different lung immune populations to identify protective cell subsets induced by MRx0004 in a model of severe asthma. Lung CD4^+^ T cell numbers were restored to untreated levels by MRx0004 treatment, in contrast to the anti-IL-17 treated group, which showed increased numbers of both CD4^+^ and CD8^+^ T cells. These distinct treatment responses support the histopathological findings on CD4^+^ T cells observed with the prophylactic dosing strategy. MRx0004 significantly increased the expression of CD44^+^ on CD4^+^ effector memory T cells. CD44 is associated with both bronchial hyperresponsiveness^60^ and resolution of inflammation in asthma^61^, depending on the specific cell subsets and whether tissues are examined during acute asthma or during the resolution stages. Further in-depth characterization of the lung tissue (e.g. hyaluronan binding activity and neuraminidase expression of these CD4^+^CD44^+^ cells) could prove informative. However, interactions between CD44 and airway smooth muscle mostly occur in the presence of pro-inflammatory mediators such as TNF-α, and we have demonstrated that MRx0004 promotes an anti-inflammatory microenvironment. CD4^+^FoxP3^+^ regulatory T cell populations were increased in the airways of MRx0004-treated mice, while reduced numbers of CD11b^+^MHCII^+^ DCs expressing CD40, CD8 or CD86 were observed. Regulatory T cells could interact directly with DCs by downregulating their surface expression of CD80/CD86, thus reducing their antigen-presenting ability and blocking the generation of allergen-specific T cell responses. Additional investigation of these specific cell subsets would further unravel the precise mechanism of action of *B*. *breve* MRx0004.

Treatment options for severe asthma remains an area of considerable unmet need, with few new drugs making it to the clinic due to lack of safety and efficacy. Despite continuing efforts to elucidate the molecular pathways responsible for driving T_H_2-low forms of asthma, blood-based biomarkers are unavailable and there are currently no approved therapeutics to act against T_H_2-low, neutrophilic associated asthma^23^. Our results show that the human gut microbiota-derived bacterial strain MRx0004 is efficacious at reducing airway inflammation in a mouse model of severe asthma. MRx0004 reduces pulmonary neutrophil infiltration without reciprocally inducing eosinophilic inflammatory influx, thus providing a novel approach to prevent and treat acute manifestations of asthma in high-risk individuals.

## Materials and Methods

### Animals

Female 7-week-old C57BL/6 mice (Charles River Laboratories, France) were randomly allocated to Sealsafe individually ventilated cages (Indulab AG). Mice were acclimated to the facility for 7 days. Mice were 8 weeks old at the initiation of the studies. Potable water and food were available ad libitum. Cage enrichment was present and daily care of the animals was performed. All experiments were run at Preclin Biosystems AG, Epalinges, Switzerland. Animal welfare regulations were observed as dictated by the official authorities of Switzerland under ordinance 455.163 of the FVO (Federal Veterinary Office) on laboratory animal husbandry, production of genetically modified animals, and methods of animal experimentation. The experimental protocol was approved by the local authorities “Service de la consommation et des Affaires vétérinaires” (SCAV) and the study protocol follows the Directive 2010/63/EU revising Directive 86/609/EEC on the protection of animals used for scientific purposes. The prophylactic study involving pre-dosing of bacteria prior to disease induction, was conducted as two independent cohorts with five animals per group each. Therapeutic studies involving bacteria dosing after disease induction were conducted as a single cohort of five animals per group.

### MRx0004 preparation

A cryo-vial of MRx0004 was thawed under sterile conditions by warming in a gloved hand and ~0.7 mL of contents injected into a Hungate tube containing 8 mL of anaerobic yeast extract-casein hydrolysate-fatty acids (YCFA) broth. Hungate tubes were then incubated at 37 °C for 14–16 h to allow the bacterial cultures to reach late log at which point they were used as treatments. A Hungate tube containing 8 mL of anaerobic YCFA was incubated at 37 °C for 14–16 h to act as vehicle.

### Severe neutrophilic asthma house dust mite (HDM) sensitization and challenge

On day (D) 0 and D7, mice in the HDM, HDM + vehicle (vehicle), HDM + MRx0004 (MRx0004) and HDM + Anti-IL-17 (Anti-IL-17) groups were sensitized with 50 µg HDM (Greer Laboratories) in phosphate-buffered saline (PBS) emulsified in an equal volume of complete Freund’s adjuvant (CFA, Chondrex Inc) and administered subcutaneously (s.c.) in a volume of 200 µL, twice over two weeks on opposite flanks. A week after the second immunisation, mice were anaesthetized by intraperitoneal (i.p.) injection with 9.75 mg xylasol and 48.75 mg ketasol per kg (Dr. E. Graeub A. G.) and then given intranasal challenges of 15 µg of HDM in a volume of 30 µL PBS starting D14 and continuing on D15, D16 and D17. On D18, mice were sacrificed by lethal i.p. injection with pentabarbitol (Streuli Pharma AG).

### Bacterial, vehicle and anti-IL-17 dosing

Preventative studies involving bacteria pre-dosing: five mice were dosed once daily by oral gavage with MRx0004 (5 × 10^8^ CFU/mouse) and another five mice were dosed with vehicle (YCFA; 400 µL/mouse) from D-14 to D17. Five mice were dosed with 12.5 mg/kg of anti-IL-17 antibody (BioX Cell, clone 17F3) on D13, D15 and D17 by i.p. injection. Five mice were treated with HDM. Two independent preventative studies were conducted using two cohorts of animals in treatment groups outlined above.

Therapeutic studies without bacteria pre-dosing: five mice were dosed once daily with MRx0004 (5 × 10^8^ CFU/mouse) from D7 to D17. Five mice were dosed with 12.5 mg/kg anti-IL-17 neutralizing antibody on D13, D15 and D17 by i.p. injection. Vehicle mice were dosed once daily with YCFA (400 µl/mouse) from D-14. Five mice were treated with HDM.

### BALF

Tracheotomy was performed immediately after euthanasia by inserting an 18 G canula (Tro-Vensite safety plus, Troge) into the trachea attached to a 1 ml syringe. BALF was obtained by flushing the lungs twice with 0.5 mL of PBS supplemented with 0.2% (v/v) Bovine serum albumin (BSA, Sigma). BALF was then transferred into a 1.5 mL Eppendorf tube and stored on ice until further processing. BALF was centrifuged at 1800 rpm for 8 min at 4 °C. Pelleted BALF cells were resuspended in PBS/2% BSA and 1 × 10^5^ cells were transferred to microscope slides using a cytospin centrifuge (800 rpm, 5 min at 4 °C) and stained with Diff-Quik staining set (Medion Diagnostics). Differential cell counts were performed based on morphological and cytochemical criteria counting 200 cells per samples in at least 2 different field of view using an Olympus BH-2 microscope.

### Measurement of inflammatory mediators in lung tissue

Right lung lobes isolated for quantification of inflammatory mediators were weighed and thereafter homogenized in 250 µL PBS supplemented with 1X protease inhibitor cocktail (Roche) using the TissueLyser system (Qiagen). Briefly, one 5 mm stainless steel bead (Qiagen) was added to each sample. Lungs were then homogenized for 3 min at 25 Hz at 4 °C. Homogenates were then spun down at 10000 rpm for 20 min at 4 °C and supernatants were collected and frozen at −20 °C. Quantification of IFN-γ, IL-1α, IL-1β, CXCL1, CCL3, CXCL2, CCL5, and TNF-α was performed using the Bio-Plex Pro Cytokine, Chemokine, and Growth Factor Assays (Bio-Rad) following manufacturer’s recommendations. Background corrected fluorescence values were converted to concentrations (pg/mL) using a standard curve. Where fluorescence values were below the detectable limit, concentrations were set at 0 pg/mL, while fluorescence above the detectable limit were excluded from the presented data analysis. IL-17A and IL-17F levels fell outside the detection limits of the assay used but have been provided in Fig. S2 of the Supplementary Information, presented using fluorescence intensity rather than pg/ml.

### Isolation of lung and histological analysis

Left lungs were fixed in 10% buffered formalin (Baker) overnight at room temperature. Tissue was processed to paraffin wax in the conventional manner and 5 µm sections cut and picked up onto Superfrost Poly-L-Lysine slides (Thermo Fisher Scientific) and dried overnight at 40 °C. Sections were dewaxed with xylene (3 times, 15 min each) and rehydrated through a series of ethanol washes before performing standard Haemotoxylin and Eosin (H&E) (Sigma Aldrich) staining. Slides were mounted with Histomount (pH 6.5–7.1, TAAB laboratories) and viewed using a Zeiss Axioskop microscope equipped with x20/0.5. Plan-NEOFLUAR objective.

From each mouse six random fields of view were digitized (these images included both bronchioles and blood vessels) using a QIMAGING camera controlled with Image Pro Plus software v6 and saved as JPEGs. Each image was then assessed for both perivascular and peribronchiolar infiltrate and allocated a single score based on the following criteria where: 0, no inflammation; 1, occasional inflammatory cells; 2, greater accumulations of inflammatory cells around the vessels 3, multifocal and even greater inflammation around vessels than that seen in grade 2; 4, severe multifocal inflammatory infiltrate^62^ by two individuals familiar with HDM lung pathology and blinded to treatment groups. The mean score was then calculated for each individual mouse.

To give an overall view of the lung sections selected slides were scanned using an Axioscan Z1 with a Hitachi HV F202scl camera equipped with a Plan Apochromat x10/0.45. objective. Scaling per pixel was 0.44 × 0.44 µm. Exposure time was 2 µs and depth of focus was 5.43 µm. Zen2 lite software was used to export images which were saved as tiff files.

### Immunocytochemistry

Serial Formalin fixed paraffin embedded (FFPE) sections prepared for histological analysis were dewaxed and rehydrated as above before performing antigen retrieval. Antigen retrieval was conducted at 90 °C for 25 minutes in Citrate buffer (Abcam). Slides were then washed twice in PBS and blocked for 30 minutes at room temperature with PBS (pH7.2) 2% BSA (Sigma Aldrich). Excess block was removed by capillary action and the sections were incubated overnight at 4 °C in a humidified chamber with 12.5 µg/mL Rabbit anti-mouse CD4 monoclonal antibody (Abcam) in PBS (pH 7.2) 2% BSA. Subsequently, sections were equilibrated to room temperature for 1 h, and washed 3 times for 10 minutes with PBS (pH 7.2). The secondary antibody was applied at 2 µg/mL Alexa Fluor™ 488 donkey anti-rabbit IgG(H + L) in PBS (pH 7.2) (Molecular probes) for 30 min in the dark, at room temperature. Slides were washed as above and counter stained with DAPI (Sigma) before mounting with Vectashield (Vector laboratories) Slides were examined using the above microscope, camera, software and x20 objective. Ten randomised fields of view were imaged using the appropriate filter sets for Alexa Fluor™ 488 and DAPI. A manual exposure time of 2 milliseconds was used throughout for the Alexa 488™ images. The digitised images were saved as JPEGs. Due to the high degree of auto-fluorescence observed in the FFPE fixed tissues, the images of CD4 positive cells were further processed in Photoshop CC 2015.1.2. The colour select tool was used to remove background from the Alexa 488 signal. Each individual image was assessed so that only clearly labelled cells were selected. The selection criteria using the colour select tool varied from a level of 98 to 130. The ‘fuzziness’ setting was kept constant. The resultant image was then overlaid on the DAPI image and flattened in Photoshop.

### Flow cytometry

#### Cell preparation

Right lung lobes were submerged in ice-cold IMDM (Iscove’s Modified Dulbecco’s media, Gibco) and cut into small pieces. Tissue was digested for 40 min at 37 °C in IMDM supplemented with 2 mg/mL collagenase type IV. Following digestion, the tissue was homogenised through 70 µm cell strainers, which were subsequently washed with 10 mL cold PBS/0.2% BSA. Cell suspensions were centrifuged (1500 rpm, 6 min, 4 °C) and pelleted cells resuspended in 1 mL PBS/0.2% BSA at which point cell counts were made using a Beckman Coulter Z2.

#### Staining for flow cytometry

100 µL of the above cell suspensions was transferred to a round bottom 96-well plate and centrifuged (3000 rpm, 1 min, 4 °C). Cell were blocked at 4 °C for 15 min using 24G2 blocking antibody (Preclin Biosystems AG) at 800 µg/mL final concentration. Cells were washed by centrifugation and resuspension with PBS/0.2% BSA. Cell were then centrifuged (3000 rpm, 1 min, 4 °C) and resuspended in 50 µl of staining solution containing appropriate primary antibodies (CD11b-PerCp-Cy5.5; CD11c-APC-Cy7; MHCII-AF700; F4/80-AF647; CD40-PE/Cy5; CD86-PE; CD80-PE/Cy7; CD4-Pacific blue; CD8-APC-Cy7; CD25-AF700; CD44-PE; all Biolegend) for 30 min at 4 °C. After two washes with PBS/0.2% BSA and one wash in PBS cells were fixed in 50 µL of BD FACS lysing solution for 15 min at 4 °C. Cells were then washed twice in PBS/0.2% BSA and resuspended in 100 µL of PBS/0.2% BSA. For intracellular staining cells were washed once more in 0.5% saponin. Cells were then incubated with primary antibody (FoxP3-AF647, Biolegend) overnight at 4 °C in 0.5% saponin. Cells were then washed once with 0.5% saponin and twice with PBS/0.2% BSA before being resuspended in 100 µL of PBS/0.2% BSA for flow cytometry analysis.

#### Flow cytometry analysis

Events were captured on a BD LSR Fortessa (BD Biosciences) equipped with 4 lasers (405 nm, 488 nm, 561 nm and 640 nm). Compensation was performed using appropriate fluorochromes and BD CompBeads (BD Biosciences). Compensation was enabled before acquiring samples using the BD FACS Diva software. The gating strategies were as follows: (a) CD4^+^ and CD8^+^ T cells: Live/dead gate, single cell gate, CD4/CD8 gate. The CD44 marker of activation on CD4^+^ and CD8^+^ T cells was expressed as MFI (Mean Fluorescence Intensity). (b) Tregs: Live/dead gate, single cell gate, CD4 Vs. CD8 gate, CD25^+^ Vs. FoxP3^+^ gated on CD4^+^ cells. (c) Dendritic cells: Live/dead gate, single cell gate, CD11c Vs. FSC-A gate, FITC (autofluorescence) Vs. F4-80 gated on CD11c^+^ cells, CD11b Vs. MHCII gated on FITC- cells. The activation markers CD40, CD80 and CD86 were measured.

### Data analysis

All graphs and statistical analyses were generated with GraphPad Prism Version 7. Data is presented as box and whisker plots with minimum and maximum and individual data points. Statistical analysis was performed by one-way ANOVA followed by Tukey’s post-test to correct for multiple comparisons, with statistical significance accepted at p < 0.05.

### Data availability

The datasets generated during and/or analysed during the current study are available from the corresponding author on reasonable request.

## Electronic supplementary material

Supplementary Information
