# Supplementary material for: Bifidobacterium breve MRx0004 protects against airway inflammation in a severe asthma model by suppressing both neutrophil and eosinophil lung infiltration
Source: Sci Rep. 2018 Aug 13;8:12024. doi: 10.1038/s41598-018-30448-z (PMC6089914; doi:10.1038/s41598-018-30448-z)
Supplement: Supplementary file 1 — Supplementary Information [file 41598_2018_30448_MOESM1_ESM.pdf]

*Bifidobacterium breve* MRx0004 protects against airway inflammation in a severe asthma model by suppressing both neutrophil and eosinophil lung infiltration.

Emma J. Raftis, Margaret I. Delday, Philip Cowie, Seánín M. McCluskey, Mark D. Singh, Anna Ettorre, Imke E. Mulder

**Supplementary Information Figure S1**

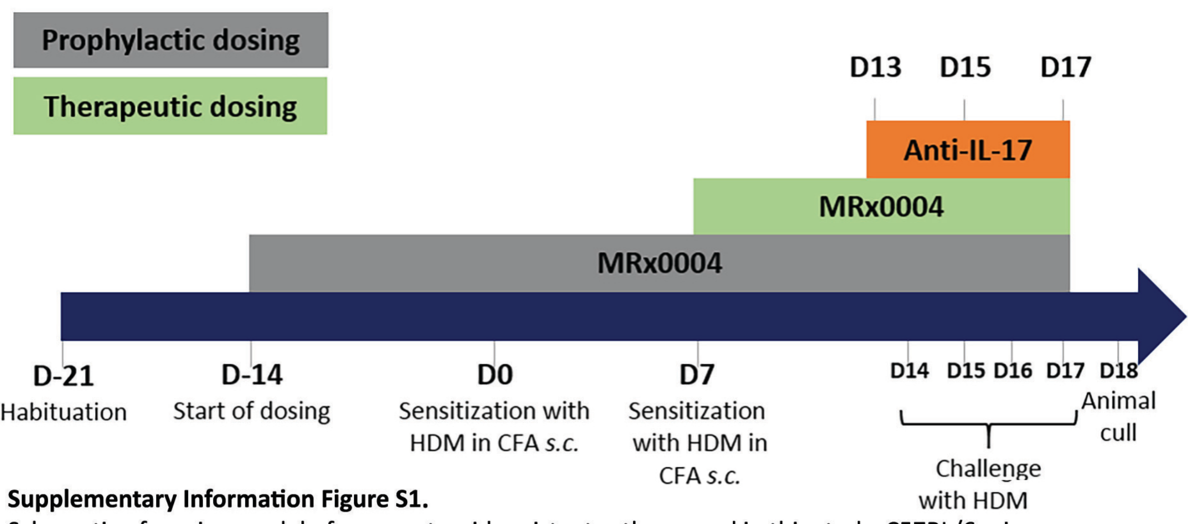


**Supplementary Information Figure S1.**
Schematic of murine model of severe steroid-resistant asthma used in this study. C57BL/6 mice were sensitized with house dust mite (HDM) in Complete Freund's Adjuvant (CFA) by subcutaneous injection at day (D) D0 and D7, and then challenged intranasally with HDM on days D14, D15, D16 and D17. See materials and methods for futher details.

**Supplementary Information Figure S2**

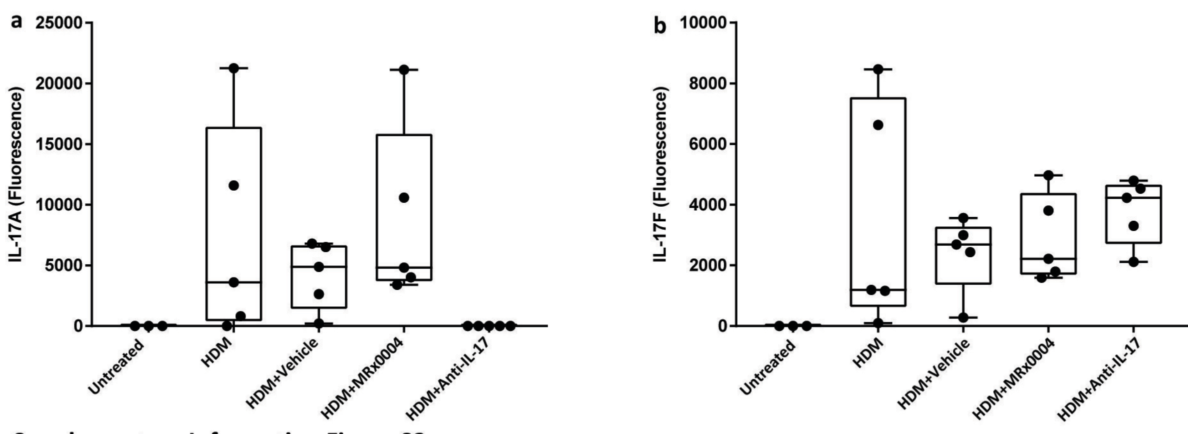


**Supplementary Information Figure S2.**
IL-17A (a) and IL-17F (b) levels in the right lungs of mice exposed to HDM followed by treatment with MRx0004, anti-IL-17 or vehicle. Untreated animals were included as a control group. Samples were collected 24h after final exposure. Results are shown as box and whisker plots with individual data points (N=3 for untreated, n=5 for all other groups) and expressed as fluorescence intensity. Capped bars illustrate the maximum and minimum data points within each treatment group.

**Supplementary Information Figure S3**

a

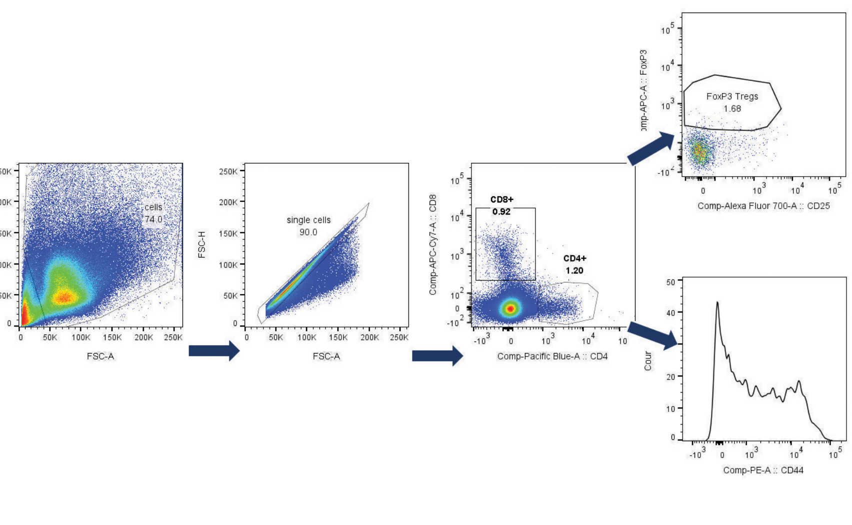


b

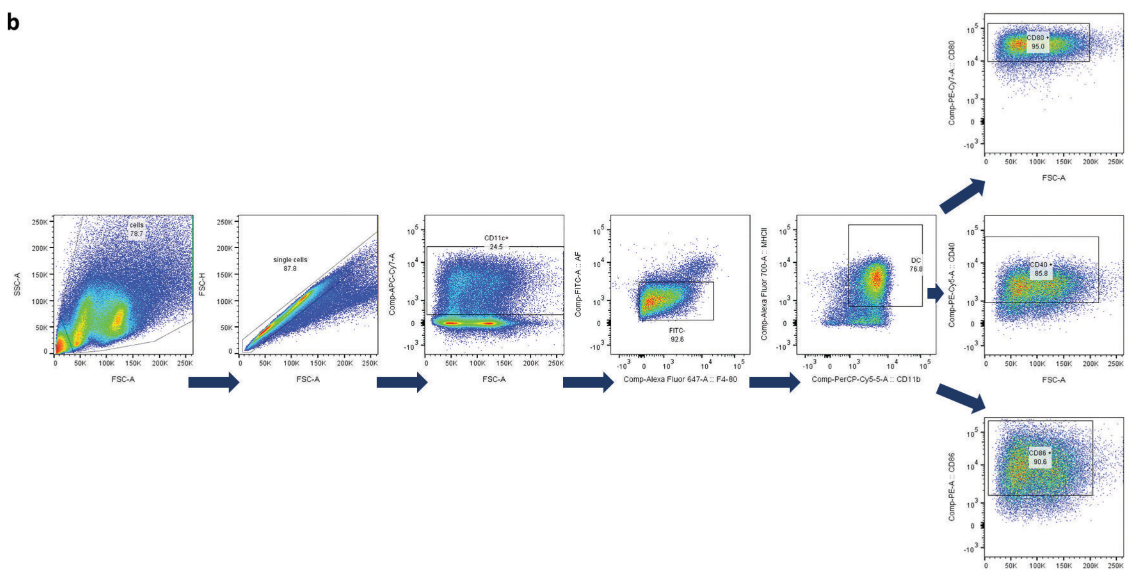


**Supplementary Information Figure S3.**

This figure illustrates the gating strategy used for the flow cytometry analysis of the different cell populations present in the lung infiltrates of mice exposed to HDM and treated with MRx0004, anti-IL-17 or vehicle. All samples were collected 24h after the final exposure. Representative dot plots from untreated mice are presented in both panels a and b. (a) T cell populations: lymphocytes were gated using the forward scatter (FSC-A) vs. the side-scatter (SSC-A) dotplot, followed by a second gate on singlet cells (FCS-H vs FSC-A). Then, CD8+ and CD4+ cells were selected to analyse the number of cells in the respective gate. From the gate defining the CD4+ population, Treg cells were identified and quantified through the expression of Foxp3 (top dotplot right hand-side). From the gate defining the CD4+ population, CD44 expression was assessed as MFI. (b) Dendritic cells (DCs). DCs population was determined using the FSC-A vs SSC-A dotplot, followed by a second gate on singlet cells and a third gate for CD11c+ cells (FSC-a vs CD11c). From the CD11c+ population, autofluorescent (AF) cells were then excluded and another gate was used to define the CD11b+MHCII+ population, defining the DCs. Expression of CD80, CD40 and CD86 markers (respective top, middle and bottom dotplots) were further analysed on the DCs gate.

**Supplementary Information Figure S4**

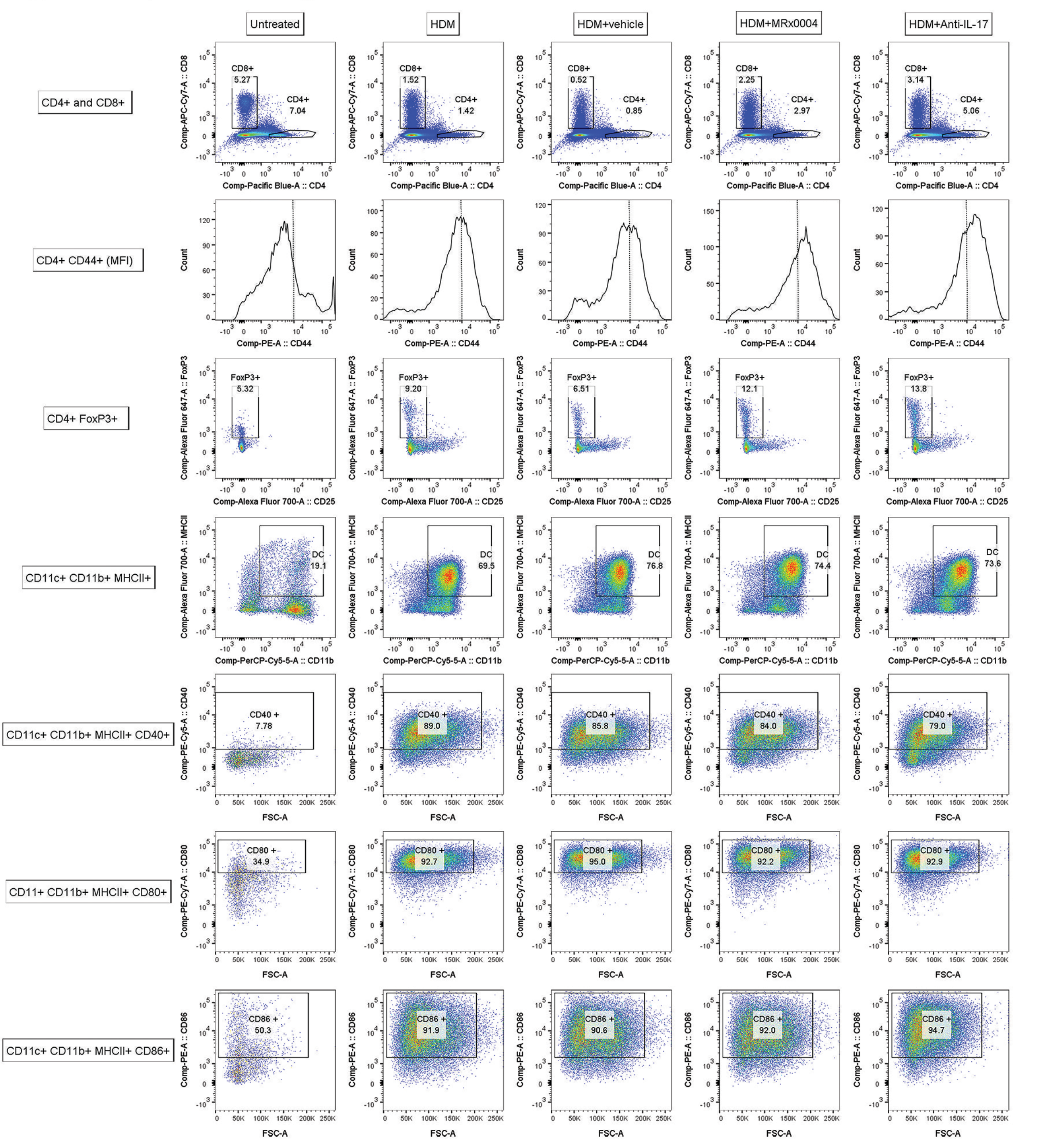


**Supplementary Information Figure S4.**
Representative dot plots of immune cell populations illustrated in Figure 5, including CD4+, CD8+, CD4+ CD44+, CD4+ FoxP3+, CD11c+ CD11b+ MHCII+, CD11c+ CD11b+ MHCII+ CD40+, CD11c+ CD11b+ MHCII+ CD80+ and CD11c+ CD11b+ MHCII+ CD86+ cells.

**Supplementary Information Table S1**

| Group | Untreated | HDM | HDM+Vehicle | HDM+MRx0004 | HDM+Anti-IL-1 |
|---|---|---|---|---|---|
| Total lung cell count | 6.98E+06 | 2.92E+07 | 2.27E+07 | 1.48E+07 | 2.54E+07 |
| %$CD4^+$ (Freq. of Parent) | 7.04 % | 1.42 % | 0.85 % | 2.97 % | 5.06 % |
| $CD4^+$ cell count | 10147 | 7771 | 9137 | 9960 | 9994 |
| Total number of $CD4^+$ cells | 4.16E+05 | 3.57E+05 | 1.68E+05 | 4.70E+05 | 9.74E+05 |
| %$CD4^+$ $FoxP3^+$ (Freq. of Parent) | 5.32 % | 9.20 % | 6.51 % | 12.1 % | 13.8 % |
| $CD4^+$ $FoxP3^+$ cell count | 540 | 715 | 595 | 1201 | 1377 |
| Total number of $CD4^+$ $FoxP3^+$ cells | 2.22E+04 | 3.05E+04 | 1.08E+04 | 5.55E+04 | 1.58E+05 |
| $CD4^+$ $CD44^+$ Geometric Mean Fluorescence Intensity | 4062 | 4955 | 5195 | 7731 | 8553 |
| %$CD8^+$ (Freq. of Parent) | 5.27 % | 1.52 % | 0.52 % | 2.25 % | 3.14 % |
| $CD8^+$ cell count | 7597 | 8363 | 5624 | 7553 | 6204 |
| Total number of $CD8^+$ cells | 3.23E+05 | 3.97E+05 | 1.07E+05 | 3.70E+05 | 6.27E+05 |
| %$CD11c^+$ $CD11b^+$ $MHCII^+$ (Freq. of Parent) | 19.1 % | 69.5 % | 76.8 % | 74.4 % | 73.6 % |
| $CD11c^+$ $CD11b^+$ $MHCII^+$ cell count | 2918 | 37693 | 37447 | 33533 | 44075 |
| Total number of $CD11c^+$ $CD11b^+$ $MHCII^+$ | 8.44E+04 | 4.41E+06 | 3.43E+06 | 2.51E+06 | 4.11E+06 |
| $CD11c^+$ $CD11b^+$ $MHCII^+$ $CD40^+$ (Freq. of Parent) | 7.78 % | 89.0 % | 85.8 % | 84.0 % | 79.0 % |
| $CD11c^+$ $CD11b^+$ $MHCII^+$ $CD40^+$ cell count | 227 | 33558 | 32137 | 28184 | 34818 |
| Total number of $CD11c^+$ $CD11b^+$ $MHCII^+$ $CD40^+$ | 6.36E+03 | 3.93E+06 | 2.94E+06 | 2.11E+06 | 3.24E+06 |
| $CD11c^+$ $CD11b^+$ $MHCII^+$ $CD80^+$ (Freq. of Parent) | 34.9 % | 92.7 % | 95.0 % | 92.2 % | 92.9 % |
| $CD11c^+$ $CD11b^+$ $MHCII^+$ $CD80^+$ cell count | 1017 | 34926 | 35581 | 30919 | 40930 |
| Total number of $CD11c^+$ $CD11b^+$ $MHCII^+$ $CD80^+$ | 2.95E+06 | 4.10E+06 | 3.27E+06 | 2.31E+06 | 3.81E+06 |
| $CD11c^+$ $CD11b^+$ $MHCII^+$ $CD86^+$ (Freq. of Parent) | 50.3 % | 91.9 % | 90.6 % | 92.0 % | 94.7 % |
| $CD11c^+$ $CD11b^+$ $MHCII^+$ $CD86^+$ cell count | 1467 | 34635 | 33944 | 30845 | 41755 |
| Total number of $CD11c^+$ $CD11b^+$ $MHCII^+$ $CD86^+$ | 4.28E+04 | 4.06E+06 | 3.11E+06 | 2.31E+06 | 3.89E+06 |

**Supplementary Information Table S1.**
Cell numbers and MFI scores that correspond to the representative dot plots illustrated in Supplementary Information Figure S4.
